# Supplementary material for: Immune-Activated B Cells Are Dominant in Prostate Cancer
Source: Cancers (Basel). 2023 Feb 1;15(3):920. doi: 10.3390/cancers15030920 (PMC9913271; doi:10.3390/cancers15030920)
Supplement: Supplementary file 1 [file cancers-15-00920-s001.zip › Suppl Figure S1.pptx]

## Slide 1
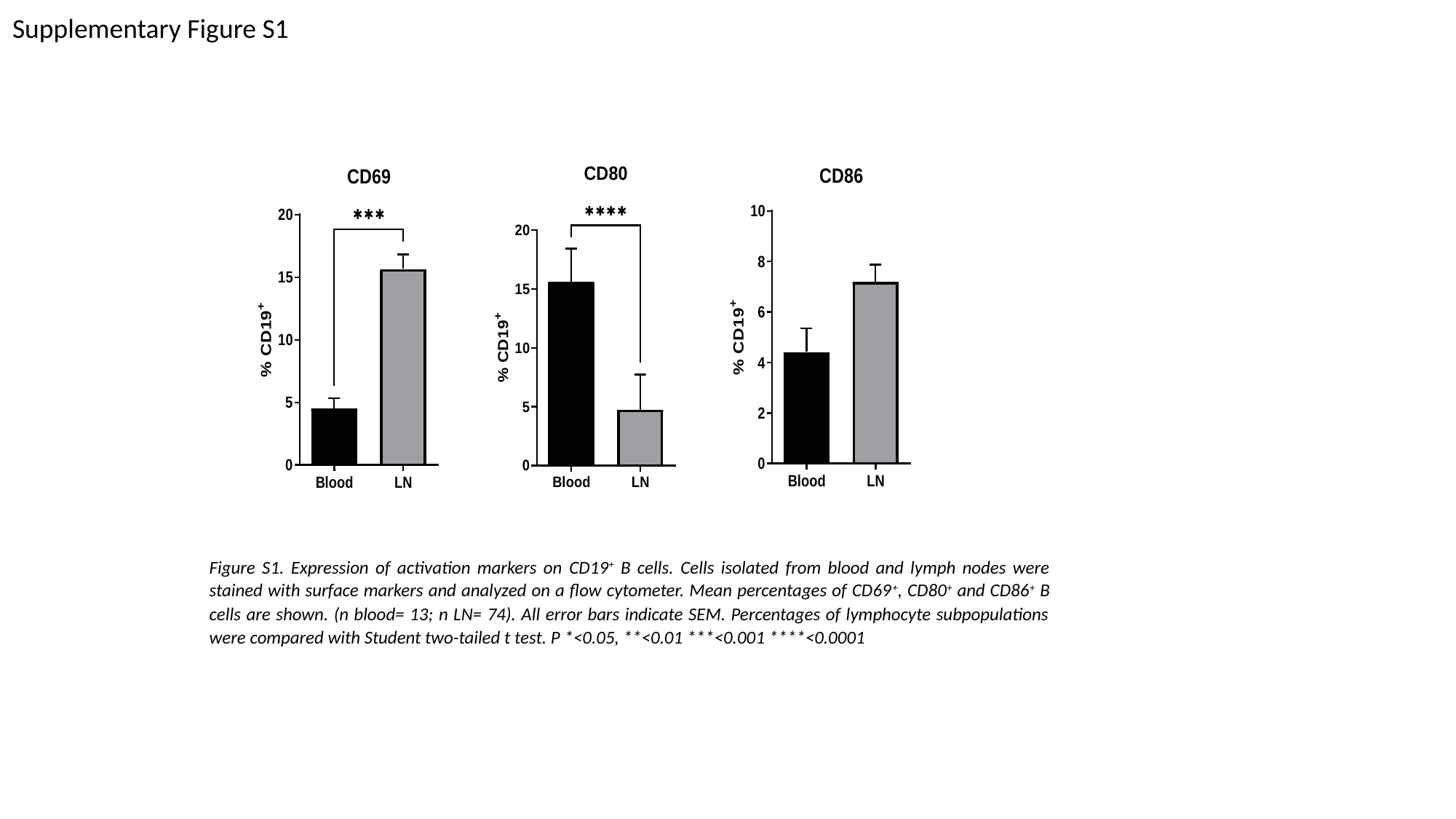

Supplementary Figure S1
Figure S1. Expression of activation markers on CD19+ B cells. Cells isolated from blood and lymph nodes were stained with surface markers and analyzed on a flow cytometer. Mean percentages of CD69+, CD80+ and CD86+ B cells are shown. (n blood= 13; n LN= 74). All error bars indicate SEM. Percentages of lymphocyte subpopulations were compared with Student two-tailed t test. P *<0.05, **<0.01 ***<0.001 ****<0.0001
