# Supplementary material for: Immune-Activated B Cells Are Dominant in Prostate Cancer
Source: Cancers (Basel). 2023 Feb 1;15(3):920. doi: 10.3390/cancers15030920 (PMC9913271; doi:10.3390/cancers15030920)
Supplement: Supplementary file 1 [file cancers-15-00920-s001.zip › Suppl Figure S3.pptx]

## Slide 1
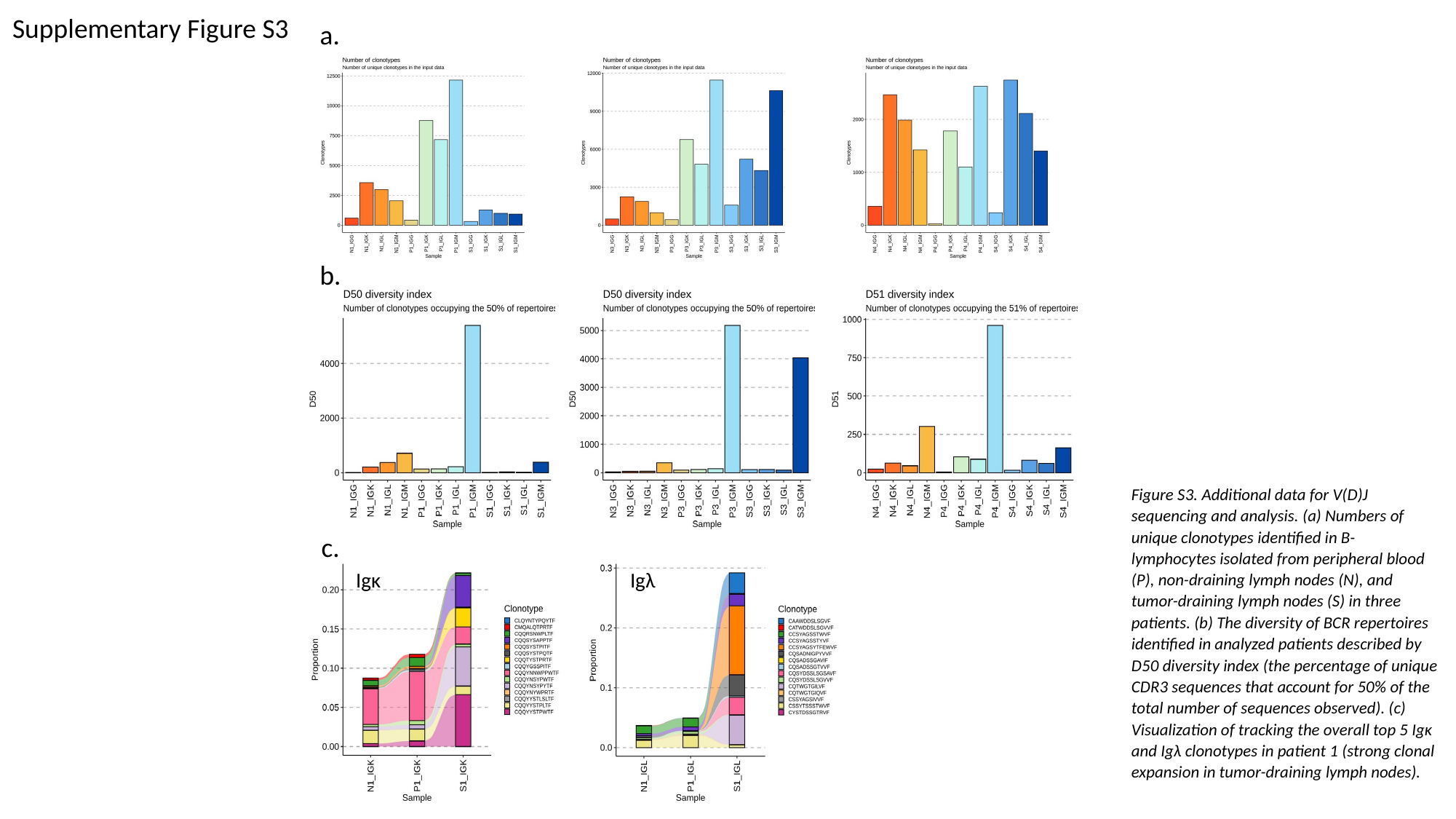

Supplementary Figure S3
a.
b.
c.
Igλ
Igκ
Figure S3. Additional data for V(D)J sequencing and analysis. (a) Numbers of unique clonotypes identified in B-lymphocytes isolated from peripheral blood (P), non-draining lymph nodes (N), and tumor-draining lymph nodes (S) in three patients. (b) The diversity of BCR repertoires identified in analyzed patients described by D50 diversity index (the percentage of unique CDR3 sequences that account for 50% of the total number of sequences observed). (c) Visualization of tracking the overall top 5 Igκ and Igλ clonotypes in patient 1 (strong clonal expansion in tumor-draining lymph nodes).
